# Developing practice guidelines to integrate physical activity promotion as part of routine cancer care: A knowledge-to-action protocol

**DOI:** 10.1371/journal.pone.0273145

**Published:** 2022-08-15

**Authors:** Isabelle Doré, Audrey Plante, Nathalie Bedrossian, Sarah Montminy, Kadia St-Onge, Jany St-Cyr, Marie-Pascale Pomey, Danielle Charpentier, Lise Pettigrew, Isabelle Brisson, Fred Saad, François Tournoux, Marie-France Raynault, Anne-Marie Mes-Masson, Lise Gauvin

**Affiliations:** 1 Centre de Recherche du Centre Hospitalier de l’Université de Montréal, Montréal, Canada; 2 School of Kinesiology and Physical Activity Sciences, Faculty of Medicine, Université de Montréal, Montréal, Canada; 3 School of Public Health, Université de Montréal, Montréal, Canada; 4 Centre Hospitalier de l’Université de Montréal, Montréal, Canada; 5 Fondation Virage, Centre Hospitalier de l’Université de Montréal, Montréal, Canada; 6 Faculty of Medicine, Université de Montréal, Montréal, Canada; 7 Centre de recherche Léa-Roback, Montréal, Canada; University of Texas Health Science Center at San Antonio, UNITED STATES

## Abstract

**Background:**

Cancer is a leading cause of disease burden worldwide and the first cause of mortality in Canada with 30.2% of deaths attributable to cancer. Given aging of the population and the improvement of prevention and treatment protocols, the number of cancer survivors is steadily increasing. These individuals have unique physical and mental health needs some of which can be addressed by integrating physical activity promotion into ongoing and long-term care. Despite the benefits of being active, delivery of PA programs for cancer patients in both clinical and community settings remains challenging. This knowledge-to-action protocol–called Kiné-Onco–aims to develop a practice guideline for the delivery, implementation, and scaling-up of cancer-specific physical activity promotion programs and services in clinical and community settings located in Québec, Canada.

**Method:**

The Kiné-Onco project involves knowledge synthesis of scientific and grey literature to establish the benefits and added value of physical activity for cancer patients and survivors, describes current practices in delivering physical activity programs, analyses quantitative data from electronic health records (EHR) of patients participating in a novel hospital-based physical activity program, collects and analyses qualitative data from patients and healthcare providers interviews about lived experience, facilitators, and barriers to physical activity promotion, outlines deliberative workshops among multidisciplinary team members to develop implementation guidelines for physical activity promotion, and summarizes a variety of knowledge transfer and exchange activities to disseminate the practice guidelines.

**Discussion:**

This paper describes the protocol for a knowledge-to-action project aimed at producing and sharing actionable evidence. Our aim is that physical activity promotion programs and services be scaled up in such a way as to successfully integrate physical activity promotion throughout cancer treatment and survivorship in order to improve the physical and mental health of the growing population of individuals having received a cancer diagnosis.

## Introduction

Cancer is a leading cause of disease burden worldwide [1] and is the leading cause of mortality in Canada with 30.2% of deaths attributable to cancer [2]. It is estimated that about 2 in 5 Canadians will be diagnosed with cancer in their lifetime and about 1 in 4 Canadians will die from cancer [2]. It was estimated that in 2021 alone, 229 200 new cases of cancer would be diagnosed in Canada and these numbers are expected to increase with the aging population that Canada and most industrialized countries are experiencing. Remarkable progress in cancer survival rates has been achieved in the past 30–40 years as a result of advances in prevention, screening, early detection, and combined modality treatment [3, 4]. This progress is reflected in the consistent decline in cancer mortality rates; in Canada, since 1988, a 37% death rate reduction in men and a 22% reduction in women were observed [2].

The growing population of cancer survivors has unique physical and mental health needs [5, 6]. For instance, consistent evidence indicates that poor physical fitness is widespread among cancer survivors and is associated with increased all-cause mortality [7], postoperative complications [8, 9], greater length of hospitalization [10], and increased hospital and health care costs [11]. The long-term cancer and cancer-treatment effects such as fatigue, pain, depressive symptoms, anxiety, fear of cancer recurrence, and cognitive impairment can develop as early as immediately following cancer diagnosis and can persist for several months or years, often becoming more severe during cancer treatment [12]. People diagnosed and treated for cancer also present a higher risk of developing comorbid conditions such as cardiovascular disease, diabetes, osteoporosis [13] as well as psychiatric comorbidities [14]. As a consequence, people being treated for cancer and cancer survivors experience high morbidity, poor functioning, and reduced health-related quality of life which lead to an increased economic burden for health care systems [15, 16].

Physical activity (PA) is demonstrated to be an effective and safe strategy to improve physical and mental health in populations diagnosed with different cancers [17–21]. Specifically, PA has benefits on physiological (e.g., muscle strength [22, 23], cardiorespiratory fitness [24], pain [25], sleep [25], physical fatigue [22, 26–29] as well as psychological (e.g., health-related quality of life [30, 31], stress, anxiety [30] and depressive symptoms [30], mental fatigue [32]) health components. Recent evidence suggests that greater involvement in PA following a cancer diagnosis is associated with significantly reduced cancer-specific and all-cause mortality [33]. In fact, because PA helps to reduce the side effects of cancer treatment, cancer patients and survivors who are physically active are more likely to be treatment observant which in turn reduces the risk of cancer recurrence and mortality [17, 34, 35]. Sedentary behaviors (i.e. waking time characterized by a low energy expenditure such as screen time or time spent in the car for transportation) [36] have been identified as an independent risk factor of cancer mortality and cancer-related symptoms or comorbidities [29] which could potentially be addressed in a strategy for PA promotion. Consistent evidence shows that PA can be safely practiced following a cancer diagnosis, respectively before, during, and after treatment [18]. Consequently, there is increasing interest in PA as an adjunct non-pharmaceutical therapy to prevent and reverse the sequelae of cancer treatment.

Since 2010, both regional (e.g. Cancer Care Ontario) [37] and international organizations (e.g. American College of Sports Medicine) [18] in North America officially recommend that people diagnosed with cancer avoid sedentary behavior and engage in regular PA. Similarly, the Clinical Oncology Society of Australia recently released a position statement endorsing the inclusion of PA as a standard of care for all cancer patients [38]. Although an increasing number of clinical trials show that PA can be practiced safely and reduces the risk of side effects, recurrence, and mortality in cancer patients [19–21, 39], limited "pragmatic" research has focused on best strategies for incorporating PA promotion into routine care, encouraging cancer-specific PA program participation, and even fewer addressing the challenges of long-term adherence to PA in community settings.

Despite cancer-specific PA guidelines [18, 40, 41], and convincing evidence of PA benefits, there are substantial challenges which preclude the implementation of PA promotion as a standard of cancer care. Delivery of PA programs for cancer patients in both clinical and community settings remains challenging and as a consequence, the vast majority of individuals living with cancer do not have the resources required to be active enough to reap benefits from PA. Even when PA programs are available, they are often unknown by health professionals and patients [42–44]. Furthermore, studies suggest that PA programs reach more women than men [45] and seem to attract patients who were already physically active at the time of diagnosis [46]. Furthermore, although the majority of oncologists are favorable to promoting PA, there is a lack of promotion tools that are well adapted to the diversity of realities experienced by patients diagnosed with different cancers [43, 47, 48]. To our knowledge, characteristics of participants engaging in real-world hospital-based cancer-specific PA programs are not known; most studies describe the sociodemographic and clinical profiles of cancer patients and survivors involved in PA intervention studies that were selected using targeted recruitment strategies. A better understanding of the characteristics and motives for engaging in existing, real-world hospital-based cancer PA programs would provide useful information to target patients and survivors that seem less likely to engage in PA and gravitate towards these programs.

An increasing number of studies [49–51], a metasynthesis [52], and a mixed methods systematic review of qualitative studies [53] have identified barriers and facilitators of PA in cancer [49, 50, 52, 53]. Most studies focus on barriers and facilitators to PA participation but do not specifically target barriers and facilitators of PA promotion along the cancer care continuum nor are they aimed at PA programs dedicated to cancer patients and survivors. Moreover, the majority of the literature has focused on patients’ experience [49, 50, 52, 53], few if any explore professionnals’ perspectives [51], and evidence from hospital administrators is scarce.

Integrating PA promotion into ongoing and long-term care is a promising innovation for addressing the physical fitness and mental health needs of cancer patients. In this project, we aim to address the gaps between knowledge and evidence regarding the PA benefits, PA programs for cancer patients and survivors, and integration of PA promotion as a routine standard of care in cancer. Effective implementation of PA as a standard of cancer care requires easy access for health professionnals and patients to the most recent evidence on PA security and benefits, identification of patient characteristics associated with PA program involvement as well as factors contributing to satisfaction and a comprehensive understanding of the barriers and facilitators to PA practice and PA promotion within the clinical setting [51, 54]. In this paper, we describe a knowledge-to-action study protocol which is designed to gather actionable evidence in support of the integration of PA promotion in the trajectory of care of individuals diagnosed with cancer and to share findings with policy-makers, managers, clinicians, patients, and the public.

### A protocol embedded within a Knowledge to Action framework

Conceptual frameworks are recommended to translate evidence into actual health interventions [55] as they provide a frame of reference that accounts for the multiple, dynamic, and interactive factors that influence effective implementation of evidence into practice [55]. The Knowledge to Action (KTA) framework [56] will guide the process of translating research evidence into practice [57]. This project aims specifically at addressing the three main steps of the Knowledge to Action process, namely 1) knowledge inquiry, 2) knowledge synthesis, and 3) production of tools and knowledge transfer products. As recommended, we are involving various stakeholders, cancer patients, health professionals, and managers, in order to tailor knowledge tools and products to needs.

### Objectives and deliverables

The ultimate deliverable of this project is to assemble the knowledge required for the production of a practice guideline allowing for delivery and implementation of cancer-specific PA promotion in clinical and community settings located in Québec, Canada. The practice guideline deliverable and the project will be built to meet the requirements of the provincial agency that develops such guidelines, namely the *Institut national d’excellence en santé et en services sociaux (INESSS)*.

The specific objectives are to:

1. Provide a review of reviews of PA safety and added value for people diagnosed with cancer;
2. Summarize PA recommendations for people diagnosed with cancer endorsed by provincial and national agencies;
3. Identify and describe the range of PA programs for cancer patients and survivors currently offered in clinical and community settings in Canada as well as internationally;
4. Gain insights into the sociodemographic and clinical profiles as well as the participation motives of cancer patients who took part in a kinesiology program offered in a hospital setting by examining HER information of patients;
5. Examine barriers and facilitators identified by patients and health professionals to the implementation of PA promotion in routine cancer care and, more specifically, participation in cancer-specific PA programs by collecting qualitative data through individual interviews;
6. Propose recommendations for the implementation and scaling-up of PA promotion and PA program participation for cancer patients and survivors in clinical and community settings in Québec by conducting interactive workshops;
7. Assemble the foundations of a practice guideline that meets the requirements of the provincial Agency responsible for approving such guidelines (INESSS) and propose tools to facilitate dissemination.

To achieve these objectives, knowledge synthesis, analysis of quantitative data from electronic health records (EHR), collection and analysis of qualitative data from patients and healthcare providers, and a variety of knowledge transfer and exchange activities will be conducted.

## Methods

### Setting

This project, henceforth called Kiné-Onco, emerged from collaborations with health professionals from the Virage Foundation (designated as Virage) offering kinesiology programs for oncology patients at the *Centre Hospitalier de l’Université de Montréal* (CHUM) which is an academic hospital with quaternary cancer care teams. The Virage kinesiology programs attracted substantial interest as many patients gravitate towards the programs. Program leaders hence expressed interest in working with a team of researchers to assemble evidence in order to translate this initiative into a larger-scale organizational innovation which might be deployed in other hospital centres. A multidisciplinary team including kinesiologists, oncologists, public health and health services researchers, managers, and policy-makers was assembled (see author list).

The CHUM is an international reference hospital centre for academic medicine and research. The CHUM welcomes annually over 8000 patients diagnosed with cancer making it the largest cancer centre in the province of Québec (Canada). The oncology healthcare team is equipped with state-of-the-art methods and instruments, provides comprehensive patient management that includes assessment, diagnosis, treatment, and support. The multidisciplinary team of the CHUM Integrated Cancer Center brings together 19 hemato-oncologists, 4 uro-oncologists, 13 surgeons (general oncology, digestive system, hepatobiliary system, gynecologic oncology, plastic), 23 nurse navigators, about 200 nurses as well as numerous other health professionals such as nutritionists, kinesiologists, and psychologists. Several foundations including Virage raise funds to offer a variety of support services to cancer patients. Starting in 2013, Virage offered a kinesiology program (see S1 Appendix for a description of the program). The kinesiologists running the Kinesiology Program also created a form to include additional information in patients’ EHRs about participation motives, individualized PA recommendations, and challenges experienced.

### Reviews of scientific and grey literature

To achieve objectives 1, 2 and 3 we will conduct reviews of peer-review scientific papers and grey literature. First, we are conducting a review of reviews of scientific publications to identify the most recent evidence on PA safety and impact of PA programs and PA involvement on both physical and psychological outcomes among people diagnosed with cancer (Objective 1) and examining the grey literature to inventory current recommendations and practices in terms of PA promotion.

With respect to the scientific literature, we are including reviews on exercise and PA undertaken before, during, and following cancer treatment by searching PubMed, Medline Google Scholar Databases, PROSPERO, and Cochrane Database of Systematic Reviews. The search strategy was applied at three different times: initially in October 2017, again in October 2018, and Spring 2020. The MeSH keywords and search strategy used in PubMed/Medline were: (((physical activity OR exercise)) AND cancer treatment) AND ("meta-analysis" OR "systematic review" OR "systematic review/meta-analysis" OR "literature review"). We are also applying the filters “last 10 years” and “Humans”. The search terms used in Cochrane Database and PROSPERO are “physical activity and cancer treatment” with the filter “in the last year”. All abstracts are screened for duplicates, only systematic reviews and meta-analysis are retained. Full texts are being downloaded and bibliography of selected publications are screened to identify other potentially relevant publications. All publications to be included in the final stage are being assessed with relevant items from the AMSTAR tool [57]. This tool comprises 11 criteria of which 6 were selected for the present project, to assess methodological quality of systematic reviews and meta-analysis. Publications not meeting the 6 criteria will be excluded. The following data are being extracted from all retained papers: number of articles included in the systematic review or meta-analysis, population and inclusion/exclusion criteria, total sample size of patients included in a meta-analysis, description of the intervention or exposure studied, comparison group, primary and secondary outcomes and main findings.

To summarize physical activity guidelines for people diagnosed with cancer (Objective 2), we are searching both scientific and grey literature and scrutinizing websites from national and international organizations from countries who provide recommendations to physicians and health professionals in oncology (e.g. American College of Sport Medicine, Cancer Care Ontario, BC Cancer, Clinical Oncology Society of Australia).

Finally, a review of exercise programs in both hospital and community settings is being conducted (Objective 3). We are searching for programs developed across six countries (Canada, Australia, New Zealand, USA, France, and the United Kingdom) using directories available on national cancer organizations’ websites, programs’ websites, and published documentation (reports, guides for patients and cancer survivors, etc.). Information on the setting (hospital, community, private), cost (free, low cost, expensive), type of program (group classes, one-on-one sessions, home training), supervision (trained exercise professionals, instructors), and content (aerobic training, strength training, full body training) are being extracted to provide a comprehensive review of program components and delivery context. In order to describe the PA programs offered in each setting, we are computing frequencies and proportions of each of the characteristics identified.

### Patient profiles of participants in the kinesiology program

To describe the profile of participants in the Virage kinesiology program (objective 4) and their “lived experience” of PA promotion, recommendations and guidance throughout their cancer experience and their participation in the program, we will perform quantitative analysis of data extracted from EHRs. Patient EHRs were populated twice by kinesiologists: once before the patient began the program (pre) and then after completion of the 8-week program (post). A smaller group of patients provided information by phone three months later which was added to data in the EHRs.

Before commencing the kinesiology program, the kinesiologists collected data about the patients’ cancer history and treatments received, health conditions, cardiovascular disease risk factors, medication, and musculoskeletal problems. The kinesiologist also asked patients to share their objectives related to participation in the PA program; objectives included: improve or maintain functional abilities, aerobic capacity, energy level, flexibility, strength, balance, decrease waist circumference, weight control, maintain glucose levels, manage hypertension, decrease dyspnea, dyslipidemia, and facilitate return to work. Patients were asked to report on the frequency, intensity, duration, and type of all current PAs (aerobic, muscular, flexibility). Information regarding current knowledge about PA and preferences for the future were also collected. Finally, selected anthropometric (e.g. height, weight, waist, arm circumference) and health indicators (e.g. cardiac frequency, blood pressure) were recorded and physical and fitness tests (e.g. balance tests, 6 min walk test, musculoskeletal tests such as sit and stand, grip strength, and joint amplitude tests) were conducted by the kinesiologist. Based on test results, the kinesiologist discussed recommendations with the patients and provided educational documentation if needed.

The post intervention information included questions about current PA and anthropometric and health indicators. Physical fitness tests were performed among those willing to ascertain changes since commencing the program. S2 Appendix provides a detailed overview of the Virage kinesiology prior to and following intervention assessment tool. The kinesiologist also inquired about any changes in disease progression and additional treatments, comorbid conditions, cardiovascular disease risk factors, medication, and musculoskeletal problems. All data collected during consultations were entered in the hospital EHR platform. Data from all patients who had participated in the PA program since its implementation in September 2013 until March 30, 2019, are being extracted by a data scientist from the CHUM Data Integration and Analysis Center (CITADEL).

We will compute descriptive statistics (frequencies, percentages, means, standard deviations, interquartile ranges) to portray the sample as a function of demographics (e.g. age, sex, etc.), cancer history and treatments (e.g. type of cancer, type of treatments, time since the end of treatments, etc.), health conditions and comorbidities, and fitness level. Histograms and Q-Q plot will be used for data visualization to illustrate the distribution of continuous variables. Box Plots will also be used to identify potential aberrant or extreme values. Then, we will compute Shapiro Wilk tests to assess normality of distributions; if the p-value is larger than 0.05, we will assume a normal distribution. Using paired T-tests, we will compare PA practice, BMI, waist circumference, cardiac frequency, and blood pressure prior to and following participation in the Virage kinesiology program. Similar analyses will be performed to compare pre-and post PA program participation results for fitness tests. All analysis will be conducted with R, Software Version 4.1.3.

### Qualitative interviews among patients and professionals

To identify facilitators and barriers to PA promotion during the cancer treatment and survivorship, and participation in cancer-specific PA programs, we are collecting data through individual interviews with i) professionals (e.g. doctors, nurses, rehabilitation specialists, nutritionists, kinesiologists, public health practitioners and managers) involved in cancer care, rehabilitation and PA services, hospital services administration, and public health and public policy and ii) cancer patients who participate in the PA program (objective 5).

The interviews with professionals aim to:

1. Explore knowledge, attitudes, and behaviors of professionals working in the health / public health sector to promote or recommend PA for cancer patients and survivors. Specific information on knowledge, attitudes and behaviors i) *required* and those representing ii) *facilitators* and iii) *barriers* to PA promotion in cancer are collected;
2. Explore information / training needs for professionals working in the health / public health sector regarding i) benefits of physical activity in the cancer care pathway, ii) availability of resources (programs, interventions, initiatives, etc.) to promote the practice of physical activity in the course of cancer care, and iii) other strategies suggested by the participants to enhance and facilitate PA promotion in cancer;
3. Identify structural facilitators or barriers (e.g., presence of infrastructure, policies relating to patient safety, well-trained staff) to the implementation of PA programs in cancer in i) hospital and ii) community settings;
4. Identify the PA promotion strategy and self-management PA strategies to i) support cancer patients and survivors to adopt and maintain PA practice in the short and long term and ii) facilitate the transition from hospital-based PA programs to programs offered in private clinics or in the community.

Interviews with cancer patients and survivors who participated in the Virage kinesiology programs between 2017 and 2019 aim at examining patient experience in transitioning from referral to the Virage kinesiology program towards home or community settings when patients undertake their PA practice autonomously. The interviews with patients aim to:

1. Identify the positive and negative experiences of patients who participated in the Virage kinesiology program;
2. Identify the facilitators and barriers (e.g., presence of infrastructure, patient safety policies, well-trained staff) to which patients have been exposed within the PA program i) in the hospital environment and ii) in community settings;
3. Identify self-management strategies spontaneously used by patients to maintain their physical activity both during participation in the Virage kinesiology program and after the end of the program.

A total of 20 professionals (5 oncologists, 4 nurses, 1 cardiologist, 1 public health practitioner, 4 kinesiologists, and 5 people with different management positions inside the healthcare system) have been identified after consultation with the research team, and are being invited to participate in interviews. A random sample of 60 participants is being drawn from the participants involved in the Virage kinesiology program in 2017–2019. To obtain in-depth data presenting multiple perspectives from patients and healthcare professionals and managers, sample size for semi-structured interviews should range between 15 and 20 participants [58]. A minimum of 15 interviews will be conducted per group (patients and healthcare professionals-managers). Given that emergence of new information decreases over time [59], data collection will end when at least two additional interviews no longer produce new information [60–62].

Research staff is making initial contacts via email and/or by phone to invite identified participants to the interviews and arrange an interview appointment. For all participants (professionals and patients), semi-structured individual interviews are performed by a trained research assistant in person, over the phone, or by video conference according to patients’ preferences. Participants receive the consent form by email using an online form or a form to print, sign, and scan; they have the opportunity to ask questions regarding the study and their participation and then provide consent before the interview. Digital audio recordings of the interviews are performed via a phone or computer recorder application and later downloaded on secured computers at the research centre. Phone recordings are then deleted. Recordings stored in secured computers will be transcribed verbatim.

The team is using the comprehensive, integrated checklist of determinants of practice checklist (TICD Checklist) as a guide for deductive thematic analysis while allowing for unexpected themes to emerge during the coding process [63]. According to Braun and Clarke [64], this method of analysis is flexible and can be used with a variety of theoretical and epistemological approaches for ‘‘identifying, analyzing, and reporting patterns (themes) within the data” (p. 79) [64]. A preliminary codebook was created during training and inter rater consensus-building based on the determinants and domains of the TICD checklist. After reviewing the research questions, four research assistants read and co-coded six excerpts and two full interviews using the preliminary codebook. Inter-rater reliability is achieved by discussing the coded excerpts until consensus is reached. The codebook will be updated by research assistants throughout the coding process and its validity is being validated during weekly research team meetings. Once research assistants reach consensus on the codebook, verbatim transcripts will be independently analyzed by three research assistants using the qualitative analysis software Dedoose Version 8.0.35.

### Scaling-up: Developing practice guidelines for PA promotion

In light of the evidence gathered in the scientific and grey literature reviews, results from the data collected on patient medical record, and findings that will emerge from in-depth analysis of professionals and patients interviews, the multidisciplinary research team, including researchers, graduate students, oncologist, kinesiologist, hospital managers, and public health decision makers, will hold 2 to 4 half-day deliberative workshops to develop specific recommendations regarding PA promotion for patients through the cancer journey, in both clinical and community settings (objective 6 and 7).

### Knowledge transfer and exchange activities

First, we propose to disseminate the resulting practice guideline and recommendations across Québec among healthcare organizations (including the Health Ministry, and the INESSS), physician-oncologists, health professionals, public health practitioners, cancer patients and survivors, and the general public. To this end, we will enlist patient partners to identify messages and tool formatting. Knowledge transfer activities will be achieved through reports, dissemination, exchanges, and production of the practice guide. Between January and June 2021, we will hold outreach events for clinicians, patients, kinesiologists, and public health actors who promote PA in communities by creating video clips of patients and health professionals and decision support tools from the study; organizing public forums on cancer and PA informed by our findings and scientific publications; publishing one or two brief "communiqués". Findings and recommendations from the current study will also be disseminated to the international scientific community through website simple language summaries and scientific publications in open access journals in French and English.

### Ethical approval

The data collection procedures were approved by the CHUM Hospital Center Research Ethics Committee; ethical approval was received on April 12, 2018 (no 17.238). Patient medical record access was approved by the institution and all data are anonymized. Participants of qualitative interviews provided written informed consent and transcripts are also anonymized. The study design complies with institutional and national research ethics regulations and has been performed in accordance with the ethical standards as laid out in the 1964 Declaration of Helsinki and its later amendments.

### Study status

The data literature review started in September 2018; the EHR data extraction was completed in July 2019. At the time of submission, the research team was finalizing qualitative interviews with both professionals and patients, running analyses on data extracted from patient EHR, and developing the blueprint for the practice guideline.

The COVID-19 pandemic affected health care systems globally. Worldwide, efforts to maintain and adapt usual health care were necessary although these transformations were not easy to implement due to limited time and resources. At the CHUM, the kinesiology team from the Virage Foundation rapidly transformed in-person PA sessions into virtual PA group sessions three times per week. To promote social interactions between participants, break social isolation, and foster social connectedness and support particularly needed in the context of COVID-19 confinement, the virtual room was available 30 minutes before and 30 minutes after PA sessions. This new virtual kinesiology service has generated great interest and increased participation from CHUM cancer patients into Virage PA programs. Although the current project was not initially planned to address this rapid transition to virtual services, it seems that there is much to learn from this rapid and so far, successful organizational transformation in the current COVID-19 context. To this end, data are being collected through the deliberation process with the multidisciplinary team to propose specific recommendations to promote PA and adapt kinesiology services in the exceptional context of a global pandemic.

## Discussion

This paper describes the protocol for a knowledge-to-action project aimed at producing and sharing actionable evidence. Our hope is that PA promotion can be scaled up in such a way as to successfully integrate PA throughout cancer treatment and survivorship in order to improve the physical and mental health of the growing population of patients having received a cancer diagnosis.

Our team recognizes that results obtained from participants mostly in one organization (hospital in a large city) would not reflect the diversity of perspectives from both patients and health professionals and managers across all regions and sites in Quebec. Given that PA programs are not widespread, it is challenging to identify individuals (both patients and caregivers) who have lived experience of either performing PA or promoting PA among cancer patients. As a result, we believe that data from this project represents a strength of the design as we capitalize on people’s lived experience to build and improve a nascent initiative. Finally, while we are in a single cancer center, the CHUM is the largest cancer center in the province of Quebec, and thus provides services and care to a broad spectrum of citizens. The size and complexity of the center provides an opportunity for understanding how different actors in the system spearheaded a variety of problems. Furthermore, we believe that working from a single, large university hospital is a good place to commence the exercise of scaling up.

## Supporting information

S1 Appendix Description of Virage’s kinesiology program, an organisational innovation. (DOCX)

S2 Appendix Information collected via the Virage kinesiology program assessment at the CHUM Integrated Cancerology Center (CICC) and then included in patient EHRs. (DOCX)

S3 Appendix Interview guides. (DOCX)
