# Supplementary material for: Developing practice guidelines to integrate physical activity promotion as part of routine cancer care: A knowledge-to-action protocol
Source: PLoS One. 2022 Aug 15;17(8):e0273145. doi: 10.1371/journal.pone.0273145 (PMC9377590; doi:10.1371/journal.pone.0273145)
Supplement: S1 Appendix — (DOCX) [file pone.0273145.s001.docx]

**APPENDIX A: Description of Virage’s Kinesiology Program, an organisational innovation**

In 1986, the Virage foundation was created to offer a variety of support services to cancer patients. The kinesiology service for oncology patients was initiated in September 2013 and currently includes a *rehabilitation program*, an *on-going treatment program*, and a *maintenance program.* In the near future a *prehabilitation program* will be offered. In 2013, the rehabilitation PA program was developed and offered to cancer patient following active treatments. It is referred to as rehabilitation because many patients become increasingly sedentary during active treatments. In 2014, programs were added for patients still undergoing active treatment. In 2017, a PA maintenance program was created for those who had completed other PA programs. Indeed, due to high demand by patients who wanted to remain active following completion of the hospital-based program, this maintenance program was incepted. Finally, an online pilot multimodal prehabilitation program is currently under development and is being launched during Fall 2020.

At any step of their cancer treatment, patients can get a referral from their oncologist, other doctors or nurses to the Virage kinesiology program. Some patients simply walk in to the Virage office which is adjacent to the CHUM oncology service waiting room (since 2018) to seek information on programs offered. Others directly call Virage after family, friends, or peers’ recommend the program. The kinesiologists from Virage then contacts the patient to schedule a complete clinical and physical evaluation. Patients who receive full clearance for PA can then choose between the various PA programs offered according to their treatment status (pre-treatment, in active treatment, post-treatment, post-rehabilitation).

For the *rehabilitation program*, patient can choose between group-based or individually-based programs. The group-based program consists of three supervised sessions per week for 8 weeks. The individually-based version of the program includes recommendations on specific exercises to do at home and advice regarding PA and healthy lifestyle in general with regular follow-up by phone from the kinesiologist. For the *in-treatment program* patients are offered a personalized exercise program with the possibility of step-into group sessions twice a week for the entore treatment duration. The *maintenance program* for those who had complete the rehabilitation program includes sessions twice a week in group setting; participants are allowed to participate for a maximum of 3 months in exchange of a small money contribution (5$ per session). All PA programs from Virage are supervised by a kinesiologist in-person for group session which take play in the Virage Gym on the hospital site or by regular phone call follow-up for home-based program. The program operates all year; in 2019, over 200 patients participated in Virage exercise programs. After completing either one of the programs, patients can be referred to PA programs in their community. To this end, kinesiologists have created a list of exercise and PA programs and services available in the Greater Montreal Area. A telephone follow-up is conducted 3 months after the end of the post-treatment program; at this time, kinesiologists offer advice to the patient at this time to facilitate his transition to PAs in his community.
