# Supplementary material for: Developing practice guidelines to integrate physical activity promotion as part of routine cancer care: A knowledge-to-action protocol
Source: PLoS One. 2022 Aug 15;17(8):e0273145. doi: 10.1371/journal.pone.0273145 (PMC9377590; doi:10.1371/journal.pone.0273145)
Supplement: S2 Appendix — (DOCX) [file pone.0273145.s002.docx]

**Appendix B – Information collected via the Virage kinesiology program assessment at the CHUM Integrated Cancerology Center (CICC) and then included in patient EHRs**

Gray text indicates informations for the evaluator

PRE-INTERVENTION ASSESSMENT

**Origin**

Patient expectations for the meeting:

Professional occupation:

Dominance: Left/Right

**Cancer history:**

- Tumor site: Date of diagnosis:
- Recurrence: Date of 1st diagnosis:
- Affected lymph nodes: Location:
- Metastasis:
- Lymphoedema:

If yes, have you consulted a specialist?

If so, what are you wearing?

**History of cancer-related treatments:**

- Surgery: Y/N + type of surgery Date:
- Chemotherapy: Y/N Date:
- Radiotherapy: Y/N Date:
- Other treatment: Y/N + list

Complications due to treatment: (infection, white blood cells, muscle pain, etc.) Y/N + list

**Other health conditions:**

- Thyroid problems:
- Dizziness: Last episode:
- Neurological problems If yes, specify:
- Balance issues:
- Stress asthma: Last episode:
- Cardiac problem:
- Others:

**Risk factors for cardiovascular disease**

- Hypertension
- Smoking
- Dislepidemia
- Abdominal / general obesity
- Diabetes: Type II
- Sedentary lifestyle
- DRS during PA (Retrosternal pain during PA - chest pain during sustained effort)
- Family history / antecedents

**Medication**:

**Musculoskeletal disorders that may influence the practice of physical activities:**

e.g. Symptoms & diagnosis, pain, accident, fall

**Referred to his attending physician:**

**Targeted objectives:**

- Improve / maintain functional abilities
- Decrease waist size
- Improve / maintain aerobic capacity
- Blood sugar control
- Improve / maintain strength
- Balanced
- Improve / maintain flexibility
- HTA
- Decrease dyspnea
- Dyslipidemia
- Back to work
- Maintenance / management of energy level
- Weight control
- Other:

**Patient action plan for goal achievement:**

**PA Antecedents**:

**Current physical activity**:

**Aerobic:**

Frequency:

Intensity:

Duration:

Type:

**Muscle type:**

Frequency:

Intensity:

Duration:

Type:

**Flexibility type:**

**Current knowledge of physical activity practice:**

Warming up

Return to calm

Perception effort scale

FITT parameters

**Preferences for the future:**

Equipment available:

Constraints:

Proposed solutions:

**MEASURES:**

HR rest:

YOUR rest:

Waist:

Weight:

BMI:

**Circumferences:**

Waist :

Mid forearm (D):

Mid forearm (L):

Mid-arm (D)

Mid-arm (G)

Others (if necessary)

**Balance test:** (Choose from one to three tests)

**Stand with your eyes closed:**

Instructions: Close your eyes and stay still for 10 seconds. Arms alongside your body, feet hip-width apart.

(4) can stand without support for 10 seconds, harmless

(3) can stand for 10 seconds, under supervision

(2) can stand for 3 seconds

(1) unable to close eyes for more than three seconds but keep balance

(0) needs help not to fall

**Stand with one foot in front of the other (tandem):**

Instructions: Place one foot directly in front of the other. If you can't, take a bigger step. To get three points, the stride length should exceed the other foot and the distance between the feet should be roughly the equivalent of a normal stride.

(4) is able to place one foot directly in front of the other, unaided, and holds the position for 30 seconds

(3) is able to take a big step without assistance and hold the position for 30 seconds

(2) is able to take a small step without assistance and hold the position for 30 seconds

(1) needs help taking a step but can hold the position for 15 seconds

(0) lose balance when taking a step or trying to stand

**Stand on one leg:**

maximum time (D) sec.

maximum time (G) sec.

Instructions: Stand on one leg for as long as possible without supporting yourself. With your arms at your sides, the lifted leg does not touch the supporting leg.

(4) can lift one leg unassisted and hold more than 10 seconds

(3) can lift one leg unassisted and hold more than 5-10 seconds

(2) can lift one leg unassisted and hold 3 seconds or more

(1) tries to lift one leg but cannot hold the position for more than 3 seconds, but remains standing

(0) cannot perform the exercise or needs help not to fall

Have the subject perform the test with each leg, note the exact score.

**Musculoskeletal tests** (Choose the 3 tests most suited to the patient's current condition)

Qualitative information (how they qualify their effort, pain felt + quantitative information)

1. Gripping force: (D) (G) Total:

2. Chair on the wall (60 seconds):

3. Sit to stand (max in 30 sec):

4. Push up (max in 30 seconds):

5. Abdominal plank (60 seconds maximum):

6. Other tests if necessary:

**Range of motion test**

Shoulder functional test (hand behind head / back - symmetry): (Normal) or (Abnormal)

Sit and reach test:

Other tests if necessary

Interpretation / analysis / explanation of results:

**Revised Goals in Kinesiology (SMART):**

**Importance of physical activity:**

**Confidence to succeed:**

**Overall impression:**

**Recommendations in AP:**

Aerobic type:

Muscle type:

Flexibility type:

Reference offered to:

Teaching / documentation provided:

Perception of effort scale

Walk log

Pedometer

Other:

**Next meeting:**

POST-INTERVENTION ASSESSMENT

**Patient expectations for the meeting:**

**Professional occupation (RAT)? :**

**Cancer history: See professional consultation note in kinesiology of the DATE**

**History of cancer-related treatments: CHECK FOR CHANGES**

- Surgical removal? Site? Date:
- End of chemotherapy treatments? Date:
- Other treatment: Type? End date :
- Complications due to treatments? (infection, white blood cells, muscle pain, etc

**Other health conditions: CHECK FOR CHANGES**

- Thyroid problems:
- Dizziness: Last episode:
- Neurological problems If yes, specify:
- Balance issues:
- Stress asthma: Last episode:
- Cardiac problem:
- Others:

**Risk factors for cardiovascular disease: CHECK FOR CHANGES**

- Hypertension
- Smoking
- Dislepidemia
- Abdominal / general obesity
- Diabetes: Type II
- Sedentary lifestyle
- DRS during PA (Retrosternal pain during PA - chest pain during sustained effort)
- Family history / antecedents

**Medication**:

**Musculoskeletal disorders that may influence the practice of physical activities:**

e.g. Symptoms & diagnosis, pain, accident, fall

**Referred to his attending physician:**

**Targeted objectives – FOLLOW-UP**

**Patient action plan for goal achievement:**

**Current physical activity**:

**Aerobic:**

Frequency:

Intensity:

Duration:

Type:

**Muscle type:**

Frequency:

Intensity:

Duration:

Type:

**Flexibility type:**

**Current knowledge of physical activity practice:**

Warming up

Return to calm

Perception effort scale

FITT parameters

**Preferences for the future:**

Equipment available:

Constraints:

Proposed solutions:

**MEASURES:**

HR rest:

YOUR rest:

Waist:

Weight:

BMI:

**Circumferences:**

Waist :

Mid forearm (D):

Mid forearm (L):

Mid-arm (D)

Mid-arm (G)

Others (if necessary)

**Balance test:** (Choose from one to three tests)

**Stand with your eyes closed:**

Instructions: Close your eyes and stay still for 10 seconds. Arms alongside your body, feet hip-width apart.

(4) can stand without support for 10 seconds, harmless

(3) can stand for 10 seconds, under supervision

(2) can stand for 3 seconds

(1) unable to close eyes for more than three seconds but keep balance

(0) needs help not to fall

**Stand with one foot in front of the other (tandem):**

Instructions: Place one foot directly in front of the other. If you can't, take a bigger step. To get three points, the stride length should exceed the other foot and the distance between the feet should be roughly the equivalent of a normal stride.

(4) is able to place one foot directly in front of the other, unaided, and holds the position for 30 seconds

(3) is able to take a big step without assistance and hold the position for 30 seconds

(2) is able to take a small step without assistance and hold the position for 30 seconds

(1) needs help taking a step but can hold the position for 15 seconds

(0) lose balance when taking a step or trying to stand

**Stand on one leg:**

maximum time (D) sec.

maximum time (G) sec.

Instructions: Stand on one leg for as long as possible without supporting yourself. With your arms at your sides, the lifted leg does not touch the supporting leg.

(4) can lift one leg unassisted and hold more than 10 seconds

(3) can lift one leg unassisted and hold more than 5-10 seconds

(2) can lift one leg unassisted and hold 3 seconds or more

(1) tries to lift one leg but cannot hold the position for more than 3 seconds, but remains standing

(0) cannot perform the exercise or needs help not to fall

Have the subject perform the test with each leg, note the exact score.

**Submaximal stress test:**

Aerobic protocol: 6 min walk test

Stop: test completed

Time / distance:

**Heart rate** pre-test:

**Blood pressure** pre-test:

**Respiratory rate** pre-test:

**Vital sign taken between 0 and 15 minutes post test:**

**Heart rate** post-test:

**Blood pressure** post-test:

**Respiratory rate** post-test:

**Effort Perception scale during and at the end of the test:**

**Comments:**

**Musculoskeletal tests** (Choose the 3 tests most suited to the patient's current condition)

Qualitative information (how they qualify their effort, pain felt + quantitative information)

1. Gripping force: (D) (G) Total:

2. Chair on the wall (60 seconds):

3. Sit to stand (max in 30 sec):

4. Push up (max in 30 seconds):

5. Abdominal plank (60 seconds maximum):

6. Other tests if necessary:

**Range of motion test**

Shoulder functional test (hand behind head / back - symmetry): (Normal) or (Abnormal)

Sit and reach test:

Other tests if necessary

Interpretation / analysis / explanation of results:

**Revised Goals in Kinesiology (SMART):**

**Importance of physical activity:**

**Confidence to succeed:**

**Overall impression:**

**Recommendations in AP:**

Aerobic type:

Muscle type:

Flexibility type:

Reference offered to:

Teaching / documentation provided:

Perception of effort scale

Walk log

Pedometer

Other:

**Next meeting:**
