# Supplementary material for: Developing practice guidelines to integrate physical activity promotion as part of routine cancer care: A knowledge-to-action protocol
Source: PLoS One. 2022 Aug 15;17(8):e0273145. doi: 10.1371/journal.pone.0273145 (PMC9377590; doi:10.1371/journal.pone.0273145)
Supplement: S3 Appendix — (DOCX) [file pone.0273145.s003.docx]

**Appendix C – Interview Guides**

**Patients – Original French Version**

**Introduction**

Bonjour!

Merci d’avoir accepté de participer à cette entrevue. Nous souhaitons échanger avec vous car vous avez participé au programme de kinésiologie de la Fondation Virage du CHUM.

Notre projet de recherche vise à mieux comprendre les bénéfices, ce qui facilite la participation mais aussi les barrières à la participation à un programme d’activité physique pour les personnes atteintes du cancer. Le but est de développer des programmes adaptés aux besoins des patients qui pourraient éventuellement être déployer à travers le Québec.

Notre équipe de recherche aimerait avoir vos impressions sur le rôle qu’a eu l’activité physique tout au long de vos soins et suivis pour le cancer, et sur le programme offert par Virage en tant que tel. Aussi, on aimerait avoir, si vous en avez, vos suggestions pour aider à faire connaître les bienfaits de l’activité physique auprès des personnes qui reçoivent un diagnostic de cancer.

Je vous rappelle que l’entrevue est confidentielle et que vous avez le droit de ne pas répondre à certaines questions. À tout moment, vous pouvez décider d’arrêter l’entrevue et de reprendre plus tard si vous le voulez.

| **Questions générales**  Pour commencer, j’aimerais savoir … | | |  |  |
| --- | --- | --- | --- | --- |
|  | Comment vous avez entendu parler du programme de kinésiologie de la Fondation Virage?  (Sonde : Est-ce qu’un médecin (oncologue, médecin de famille, autre) ou un autre professionnel de la santé vous a recommandé de faire de l’activité physique après votre diagnostic de cancer ? Si *OUI*, noter à quel moment et quel type de professionnel (Si plusieurs, tous les noter) et quel type d’activité). | |  |  |
| **Participation au programme**  J’aimerais maintenant vous poser des questions rapides concernant vos habitudes d’activité physique **avant** votre participation au programme de la Fondation Virage. | | |  |  |
|  | Faisiez-vous de l’activité physique avant de recevoir votre diagnostic de cancer? | |  |  |
|  | Si *OUI* noter:   1. Type d’activités pratiquées 2. Fréquence (par semaine) 3. Durée 4. Intensité   Léger : yoga, bowling, golf, marche  Modérée : marche rapide, badminton, danse, baseball  Élevée : course, soccer, vélo longue distance   1. Lieux 2. Coûts liés aux activités | |  |  |
|  |  | |  |  |
|  | À quel moment avez-vous débuté le programme à la Fondation Virage? Pendant, après les traitements? | |  |  |
|  | Si *APRÈS* les traitements, noter   1. Combien de temps après les traitements | |  |  |
|  | Si *PENDANT* les traitements, passer à 6. | |  |  |
|  | Est-ce qu’un médecin ou un professionnel de la santé vous a déconseillé de faire de l’AP après votre diagnostic de cancer ? Si oui, pourquoi ? | |  |  |
|  | Qu’est-ce qui vous a incité à participer au programme au début? (Sondes : accessibilité, proches, md, coûts, bouche à oreille positif, etc.) | |  |  |
| **Freins ou leviers à la participation au programme** | | |  |  |
|  | Qu’est-ce qui vous a motivé à participer aux séances tout au long du programme avec Virage? | |  |  |
|  | Est-ce qu’il y a des éléments qui ont rendu votre participation plus difficile? | |  |  |
|  | Est-ce que vous auriez été intéressé à participer à un programme d’activité physique dans votre communauté pour les gens qui ont un cancer (à proximité de chez vous, dans un centre communautaire, un gym/centre sportif, un YMCA)? | |  |  |
|  | Au contraire, est-ce qu’il y a des éléments qui vous décourageraient à participer à un programme d’activité physique dans votre communauté plutôt qu’à l’hôpital comme le programme de Virage ? | |  |  |

| **Effets positifs et négatifs du programme**   1. Pouvez-vous **me décrire les impacts/effets** que le programme a eu pour vous ? (Au besoin : Sur votre niveau d’activité physique, votre santé physique, psychologique, etc.) Ce peut être des effets positifs ou négatifs ou les deux.   Sondes à explorer : débuter/reprendre l’AP, impact sur les symptômes du cancer et des traitements, activités quotidiennes, bien-être physique/psychologique, soutien social, etc. Négatif : anxiété, fatigue, découragement, douleur, etc.   1. Aviez-vous des craintes ou préoccupations avant de participer au programme ? Si oui, lesquelles ? Ont-elles changé durant le programme ? Qu’est-ce qui a aidé à les diminuer (le cas échéant) ? 2. Avez-vous continué à faire de l’activité physique après le programme ?   Si oui : plus ou moins ou idem qu’avant le diagnostic de cancer et **qu’est-ce qui vous a motivé à continuer** (sondes : programme spécial, lieux, entraîneur, impact du programme, etc.) ?   1. Qu’est-ce qui peut rendre ça plus difficile, parfois? Qu’est-ce qui aurait pu vous aider à continuer à faire de l’activité physique? Quelles sont les stratégies personnelles que vous avez utilisées pour rester actif?   (sondes : craintes, motivation, coûts, accessibilité, manque d’information, fixation d’objectifs personnels, utilisation d’un iWatch ou fitbit, faire de l’exerice avec un/e ami ou proche, achat de nouveaux équipements tels des chaussures ou vêtements) ? | |
| --- | --- |
| **Appréciation et suggestions** | |
| 14. | Qu’est-ce que vous avez **le plus apprécié** du programme? |
| 15. | Qu’est-ce que vous avez **le moins apprécié** du programme?  (sondes : accessibilité, personnel, groupes, activités, etc.) |
| 16. | Est-ce que vous **recommanderiez ce programme** à un proche qui aurait eu un diagnostic de cancer semblable au vôtre ?  Pourquoi, pourquoi pas ? |
| 17. | Si un programme d’AP vous avait été proposé **avant de débuter les traitements** contre le cancer (dès le diagnostic), auriez-vous été intéressé à y participer?  Pourquoi, pourquoi pas ? |

| **Conclusion**  Si vous le permettez, j’aimerais terminer en vous posant quelques questions reliées à votre diagnostic de cancer et sur profil sociodémographique. | |
| --- | --- |
| 18. | Occupez-vous un emploi actuellement? |
|  | Si *OUI*:   1. Titre de l’emploi 2. Temps plein ou temps partiel 3. Est-ce que la maladie ou les traitements ont occasionné un changement dans votre statut d’emploi ? |
|  | Si *NON* :   1. Noter l’occupation (retraite, chômage, aide sociale, autre) |
| 19. | De quelle origine ethnique êtes-vous ? (Ex : Caucasien(ne), afro-américain(e), indienne, etc.) |
| 20. | Quel type de cancer avez-vous eu? |
| 21. | Quels types de traitement avez-vous reçu? |
| 22. | Avez-vous autre chose à ajouter sur le programme de la Fondation Virage ou sur l’activité physique ? |

**C’était ma dernière question pour vous. Est-ce qu’il y a autre chose que vous souhaiteriez rajouter?**

**J’aimerais vous remercier d’avoir partagé votre temps et votre expérience avec nous, ça nous sera très utile pour émettre des recommandations.**

**Patients – English Version**

**Introduction**

Hi!

Thank you for agreeing to participate in this interview. We want to chat with you because you participated in the kinesiology program of the CHUM Virage Foundation.

Our research project aims to better understand the benefits of this type of program, as well as the facilitators and barriers to participation in a physical activity program specific for people diagnosed with cancer. The goal is to develop programs adapted to patients’ needs that could eventually be deployed throughout the province of Québec.

Our research team would like to have your impressions on the role that physical activity has played throughout your cancer care and follow-up, and on the program offered by Virage itself. Also, we would like to hear your suggestions, if any, to help promote the benefits of physical activity among people who receive a cancer diagnosis.

I would like to remind you that this interview is confidential and that you have the right not to answer certain questions. At any given moment, you can decide to stop the interview completely or to resume later if you wish to.

**General questions**

To begin with, I would like to know…

1. How did you hear about the Virage Foundation kinesiology program?

(Probes: Has a doctor (oncologist, family doctor, other) or another health professional recommended physical activity practice to you after you received a cancer diagnosis? If YES, note when, by what type of professional (if many, note all) and what type of activity).

**Program participation**

Now, I would like to ask you some questions about your physical activity habits BEFORE your participation in the Virage Foundation program:

1. Were you doing physical activity before receiving your cancer diagnosis?

If YES, note:

1. Type of activities
2. Frequency (per week)
3. Duration
4. Intensity

Low: yoga, bowling, golf, walking

Moderate: fast walking, badminton, dance, baseball

High: jogging, soccer, biking long distances

1. Location
2. Cost of activities
3. When did you start the program at the Virage Foundation? During or after treatment?

If AFTER treatments, note:

1. How long after treatment

If DURING treatment, skip to question 6.

1. Did a doctor or a health professional ever advise you against doing physical activity after your cancer diagnosis? If yes, why?
2. What encouraged you to take part in the program at first? (Probes: accessibility, close relatives, doctors, costs, positive word of mouth, etc.)

**The following questions concern facilitators or barriers to participation in the program:**

1. What motivated you to participate in the sessions throughout the program?
2. Were there elements that made your participation more difficult?
3. Would you have been interested in taking part in a physical activity program in your community for people diagnosed with cancer (close to your home, in a community center, a gym/sport center, a YMCA, etc.)?
4. On the contrary, are there elements that would discourage you from taking part in a physical activity program in your community rather than at the hospital, like Virage program?

**The following questions concern positive and negative effects of the program**:

1. Can you describe the impacts/effects the program has had on you? (Probes: on your physical activity level, on your physical/mental health, etc.). These can be positive, negative or both

Positive probes: start/resume physical activity, impact on cancer or treatment-related symptoms, daily activities, physical/psychological well-being, social support, etc.

Negative probes: anxiety, fatigue, discouragement, pain, etc.

1. Did you have any concerns or fears before taking part in the program? If so, what were they?

Did they change during your participation in the program? What (if anything) helped to reduce them?

1. Did you continue to do physical activity after the program? If yes: more, less or the same as before your cancer diagnosis and what motivated you to continue? (Probes: special program, location, coach, impact of the Virage program, etc.)
2. What can make it more difficult sometimes? What could help you continue practicing physical activity? What personal strategies do you use to stay active?

(Probes: concerns, fears, motivation, cost, accessibility, lack of information, setting personal goals, using an iWatch or Fitbit, practicing physical activity with family or friends, buying new equipment like shoes or clothes, etc.)

**The following questions concern your appreciation and your suggestions for the program**:

1. What did you like the most about the program?
2. What did you like the least about the program?

(Probes: accessibility, staff, groups, activities, etc.)

1. Would you recommend this program to a close relative who received a cancer diagnosis similar to yours?

Why? Why not?

1. If a physical activity program had been offered to you BEFORE you started cancer treatment (upon diagnosis), would you have been interested in participating?

Why? Why not?

**Conclusion**

If it’s alright with you, I would like to conclude the interview by asking you some questions regarding your cancer diagnosis and your sociodemographic profile.

1. Are you employed at the moment?

If YES:

1. Job title
2. Full time or part-time
3. Has cancer or its treatments caused any changes to your employment status?

If NO:

1. Occupation (retired, student, unemployment, social welfare, etc.)
2. What is your ethnicity? (Caucasian, Afro-American, Indian, etc.)
3. What type of cancer did you have?
4. What type of treatment did you receive?
5. Do you have anything else to add about the Virage Foundation or about physical activity?

This was my last question for you. Is there anything else you would like to add?

I would like to thank you for your time and for sharing your experience with me, it will help us tremendously in developing physical activity recommendations for cancer patients.

**Health professinnals and Managers – Original French Version**

**Introduction**

Merci d’accepter de participer à cette entrevue.

Nous souhaitons recueillir vos perceptions/idées relativement à la promotion de l’activité physique auprès de patients ayant reçu un diagnostic de cancer dans le cadre d’un projet de recherche qui vise à mieux comprendre : 1) les bienfaits des programmes d’activité physique offerts aux personnes atteintes de cancer, 2) les éléments qui facilitent/limitent l’implantation de ces programmes et 3) la façon de favoriser une participation sécuritaire des patients à ces programmes. Les informations recueillies serviront à orienter les pratiques et programmes dans le réseau de la santé ou dans la communauté à travers le Québec (et potentiellement plus largement).

Je vous rappelle que l’entrevue est confidentielle et que vous avez le droit de ne pas répondre à certaines questions si vous ne le souhaitez pas. À tout moment, vous pouvez décider d’arrêter l’entretien et de reprendre plus tard si vous le souhaitez.

| **Questions générales**  J’aimerais débuter par des questions plus générales. | | | |
| --- | --- | --- | --- |
|  | Quel est votre titre d’emploi? |  |  |
|  | Si *direction* et *kinésiologues* de la *Fondation Virage* ou *direction/gestionnaires de l’hôpital*  Sauter 12 et 13. |  |  |
|  | Si *autre professionnel* non impliqué dans Virage ou la gestion de l’hôpital  Sauter 8 à 11. |  |  |
| **Attitudes et connaissances sur l’AP**  J’aimerais dans un premier temps vous poser quelques questions en ce qui concerne vos connaissances, vos attitudes et votre pratique clinique habituelle en lien avec la pratique d’activité physique chez les personnes ayant reçu un diagnostic de cancer/pour la clientèle en oncologie. | |  |  |
|  | À la lumière de votre pratique clinique et de vos connaissances des écrits scientifiques, comment décrirez-vous les bénéfices de l’AP pour les personnes ayant reçu un diagnostic de cancer? |  |  |
|  | Si OUI, noter lesquels :   1. Psychologiques 2. Santé physique 3. Fatigue 4. Rétention du traitement |  |  |
|  | Si NON, passer à 3. |  |  |
|  | Abordez-vous la pratique d’activité physique avec vos patients dans votre pratique clinique? |  |  |
|  | Si OUI,   1. De quelle façon? 2. Mots clés 3. Stratégies proposées aux patients |  |  |
|  | En ce qui a trait à l’idée de recommander l’AP pour vos patients, à quel point vous sentez-vous à l’aise ou outillé? |  |  |
|  | Si *BIEN* OU *ASSEZ BIEN*, passer à 5. |  |  |
|  | Si *PEU* :   1. Quels éléments vous aideraient à vous sentir plus à l’aise/mieux formé/mieux outillé pour recommander l’activité physique avec vos patients? | |  |
|  | Est-ce que recommander l’AP auprès de patients ayant reçu un diagnostic de cancer devrait faire l’objet de collaboration interprofessionnelle? | |  |
|  | Si OUI :   1. Quels professionnels de la santé devraient être impliqués dans cette tâche? 2. Quels rôles devraient-ils prendre? | |  |
|  | Si NON :   1. À qui revient la responsabilité de recommander l’AP? 2. Pourquoi? | |  |
|  | Avez-vous reçu des témoignages de patients qui pratiquent de l’AP relativement à l’apport que cela a eu pour eux? | |  |
|  | Inversement, avez-vous reçu des témoignages de patients indiquant que l’AP leur avait été défavorable? | |  |
|  | Si *OUI* à 6 ou 7 :   1. Pouvez-vous décrire la teneur de ces témoignages? | |  |
| **[Pour professionnels et gestionnaires de la Fondation Virage/de l’hôpital seulement]**  **Implication dans le programme Virage**  J’aimerais maintenant connaître votre implication dans le développement, la mise en œuvre du programme de kinésiologie de la fondation Virage qui offre des programmes d’activité physique (programmes individuels et en groupe - principalement) aux patients en oncologie du CHUM. | | |  |
|  | Quel a été/est votre rôle dans le développement, la mise en œuvre ou la promotion du programme de la fondation Virage? | |  |
|  | Depuis combien de temps êtes-vous impliqué dans ce programme? | |  |
|  | Quelles sont/ont été les principales tâches liées à votre implication dans ce programme? | |  |
|  | Croyez-vous que le programme de kinésiologie de la fondation Virage répond à un besoin? Doit être maintenu ? Devrait être bonifié ? Devrait être déployé encore plus largement pour rejoindre davantage de patients? | |  |
| **[Pour professionnels n’œuvrant pas à la Fondation Virage seulement]**  **Connaissance du programme et référencement**  Nous aimerions maintenant discuter avec vous du programme de kinésiologie de la fondation Virage qui offre des programmes d’activité physique (programmes individuels et en groupe - principalement) aux patients en oncologie du CHUM. | | |  |
|  | Connaissez-vous le programme d’AP de la fondation Virage du CHUM? | |  |
|  | Si OUI   1. Comment en avez-vous entendu parler? 2. Référez-vous des patients au programme d’AP de la Fondation Virage? | |  |
|  | Si OUI,   1. Chez vos patients qui participent à ce programme, quels effets avez-vous pu remarquer? | |  |
|  | Si NON, passer à 13. | |  |
|  | Connaissez-vous d’autres programmes d’AP pour les patients atteints de cancer? Au CHUM? Ailleurs? | |  |
|  | Si OUI, noter :   1. Lesquels 2. Référez-vous des patients à ces autres programmes?    1. Pour quelles raisons? | |  |
|  | Si NON, passer à 14. | |  |
| **Freins et leviers à l’implantation des programmes d’AP comme celui offert par la Fondation Virage**  Maintenant, j’aimerais connaître votre opinion en ce qui a trait aux freins et aux leviers à l’implantation et à la mise en œuvre de programme d’AP pour les personnes qui reçoivent un diagnostic de cancer. | | |  |
|  | À votre avis, quels sont les principaux freins à l’implantation de programmes d’activité physique en milieu hospitalier pour les personnes atteintes de cancer? | |  |
|  | Utiliser les sondes suivantes après la réponse initiale :   1. Manque de support de l’équipe soignante 2. Connaissances sur l’AP 3. Manque de temps pour en parler aux patients 4. Financement, ressources et infrastructures 5. Formation des intervenants 6. Manque de synergie entre les groupes d’intervenants | |  |
|  | À votre avis, quels sont les principaux leviers facilitant l’implantation d’un programme d’activité physique en milieu hospitalier pour les personnes atteintes de cancer? | |  |
|  | Utiliser les sondes suivantes après la réponse initiale:   1. Caractéristiques du programme 2. Support de l’équipe soignante 3. Formation transdisciplinaire des intervenants 4. Environnement sécuritaire et facile d’accès 5. Partenariats 6. Intégration du programme dans le service d’oncologie | |  |
|  | Comment décririez-vous votre niveau de confort à référer vos patients à un programme d’activité physique adapté pour les personnes atteintes de cancer dans la communauté (à l’extérieur de l’hôpital, dans un gym privé, un centre communautaire)? | |  |
|  | Si OUI,   1. A votre avis, quelles seraient les conditions nécessaires pour référer vos patients à ces programmes dans la communauté?   Noter des informations sur   - 1. Formation reconnue par les associations professionnelles en AP   2. Évaluation clinique des aptitudes physiques   3. Référence médicale préalable | |  |
|  | Si NON,   1. Pour quelles raisons? | |  |
|  | À votre avis, qui devrait être responsable de payer des abonnements dans des centres de conditionnement physique dans la communauté? (RAMQ, assurances privées, autres) | |  |
|  | Croyez-vous qu’il serait facilitant de développer des programmes d’AP pour la préhabilitation* des patients afin qu’ils aient accès à un programme d’AP dès le diagnostic, avant d’entreprendre les traitements? | |  |
|  | *Préhabilitation : programme d’AP entre le diagnostic et le début des traitements pour améliorer la condition physique des patients afin que ceux-ci soient mieux préparés à subir les traitements. | |  |
| **Après le programme (NE PAS POSER AUX ONCOLOGUES)**  Enfin, j’aimerais connaître votre avis quant aux possibles stratégies ou composantes d’interventions qui permettraient de favoriser le maintien de l’AP / comportement actif à long terme, une fois que les participants ont complété un programme d’AP (d’une durée de 3 à 6 mois généralement) offert en milieu hospitalier comme celui de la Fondation Virage. | | |  |
|  | Selon vous, quelles stratégies permettent de favoriser la poursuite de saines habitudes de vie, et plus spécifiquement la pratique d’activité physique, une fois le programme terminé, dans la communauté? | |  |
|  | De quels outils/stratégies/pratiques aurait-on besoin pour faciliter la transition des patients vers la pratique d’activité physique dans la communauté? | |  |
| **Conclusion**  Pour terminer, j’aurais une dernière question d’ordre démographique à vous poser. | | |  |
|  | À quelle tranche d’âge appartenez-vous? | |  |
|  | 1. 25-34 ans 2. 35-49 ans 3. 50-64 ans 4. 65 ans et plus | |  |
|  | Avez-vous d’autres idées que vous aimeriez partager sur l’activité physique pour les personnes ayant reçu un diagnostic de cancer? | |  |

**Health professinnals and Managers – English Version**

**Introduction**

Thank you for agreeing to participate in this interview.

We are interested in gathering your perceptions/ideas regarding the promotion of physical activity among patients who have been diagnosed with cancer as part of a research project that aims to better understand: 1) the benefits of physical activity programs offered to people with cancer, 2) the elements that facilitate/limit the implementation of these programs and 3) the way to promote safe patient participation in these programs. The information collected will be used to guide practices and programs in the health care system or in the community across Quebec (and potentially more widely).

I would like to remind you that the interview is confidential and that you have the right not to answer certain questions if you do not wish to do so. At any time, you can decide to stop the interview and resume later if you wish.

**General Questions**

I would like to start with more general questions.

- - - 1. What is your job title?

If the manager and kinesiologists of the Virage Foundation or the managers of the hospital

Skip 12 and 13.

If other professional that is not involved in Virage or hospital management

Skip 8 to 11.

**Attitudes and knowledge about PA**

I would first like to ask you a few questions regarding your knowledge, your attitudes and your usual clinical practice related to physical activity practice among people who have been diagnosed with cancer/for oncology clients.

- - - 1. In light of your clinical practice and your knowledge of the scientific literature, how would you describe the benefits of PA for people diagnosed with cancer?

If YES, note which ones:

1. Psychological
2. Physical health
3. Fatigue
4. Treatment retention

If NO, skip to 3.

- - - 1. Do you discuss physical activity practice with your patients in your clinical practice?

If YES,

1. In what way?
2. Key words
3. Strategies offered to patients
   - - 1. In terms of ​​recommending PA for your patients, how comfortable or equipped do you feel?

If GOOD OR FAIRLY GOOD, go to 5.

Il little :

1. What elements would help you feel more comfortable/better trained/better equipped to recommend physical activity to your patients?
   - - 1. Should recommending PA to patients diagnosed with cancer be the subject of interprofessional collaboration?

If YES :

1. Which health professionals should be involved in this task?
2. What roles should they take?

If NO :

1. Whose responsibility is it to recommend PA?
2. Why?
   - - 1. Have you received testimonials from patients who practice PA about how it has helped them?

- - - 1. Conversely, have you received testimonials from patients indicating that PA has been unfavorable to them?

If YES to 6 or 7:

1. Can you describe the content of these testimonies?

**[For professionals and managers of the Virage Foundation/hospital only]**

**Involvement in the Virage program**

I would now like to know your involvement in the development and implementation of the Virage Foundation's kinesiology program, which offers physical activity programs (individual and group programs - mainly) to oncology patients at the CHUM.

- - - 1. What was/is your role in the development, implementation or promotion of the Virage Foundation program?
      2. How long have you been involved in this program?
      3. What are/were the main tasks related to your involvement in this program?
      4. Do you believe that there is a need for the Virage Foundation's kinesiology program? Should it be maintained? Should it be improved? Should it be expanded to reach more patients?

**[For professionals not working at the Virage Foundation only]**

**Program awareness and referral**

We would now like to discuss with you the kinesiology program of the Virage Foundation, which offers physical activity programs (individual and group programs - mainly) to oncology patients at the CHUM.

- - - 1. Are you familiar with the PA program of the Fondation Virage du CHUM?

If yes

1. How did you hear about it?
2. Do you refer patients to the Fondation Virage PA program?

If YES,

a) What effects have you noticed in your patients who participated in this program?

If NO, skip to 13.

- - - 1. Do you know of any other PA programs for cancer patients? At the CHUM? Elsewhere?

If YES, note:

1. Which programs?
2. Do you refer patients to these other programs?
   - - - 1. For what reasons?

If NO, skip to 14.

**Barriers and facilitators to the implementation of PA programs such as the one offered by the Virage Foundation**

Now, I would like to know your opinion regarding the barriers and facilitators to the implementation and delivery of PA programs for people who are diagnosed with cancer.

- - - 1. In your opinion, what are the main barriers to the implementation of physical activity programs in hospitals for people with cancer?

Use the following probes after the initial response:

1. Lack of support from the healthcare team
2. Knowledge about PA
3. Lack of time to talk to patients
4. Funding, resources and infrastructure
5. Training of health professionals
6. Lack of synergy between health professionals’ groups
   - - 1. In your opinion, what are the main facilitators of the implementation of a physical activity program in a hospital setting for people with cancer?

Use the following probes after the initial response:

1. Characteristics of the program
2. Support from the health care team
3. Transdisciplinary training of health care professionals
4. Safe and accessible environment
5. Partnerships
6. Integration of the program into the oncology department
   - - 1. How would you describe your level of comfort in referring your patients to a physical activity program adapted for people with cancer in the community (outside the hospital, in a private gym, community centre)?

If YES,

1. In your opinion, what would be the necessary conditions to refer your patients to these programs in the community? Record information about
2. Training recognized by professional associations in PA
3. Clinical assessment of physical abilities
4. Prior medical referral

If NO,

1. For what reasons?
   - - 1. In your opinion, who should be responsible for paying memberships for community fitness centres? (RAMQ, private insurance, others)
       2. Do you think it would be easier to develop prehabilitation* PA programs for patients so that they have access to a PA program as soon as they are diagnosed, before starting treatment?

*Prehabilitation: PA program between diagnosis and the start of treatment to improve the physical condition of patients so that they are better prepared to undergo treatment.

**After the program (DO NOT ASK ONCOLOGISTS)**

Finally, I would like to hear your thoughts on possible strategies or components of interventions that would make it possible to promote the maintenance of PA / active behavior in the long term, once the participants have completed a PA program (typically 3 to 6 months in duration) offered in a hospital environment such as that of the Virage foundation.

- - - 1. In your opinion, what strategies make it possible to promote the maintenance of healthy lifestyles, and more specifically the practice of physical activity, once the program is completed, in the community?
      2. What tools/strategies/practices would be needed to facilitate the transition of patients to physical activity practice in the community?

**Conclusion**

Finally, I have one last demographic question for you.

- - - 1. What age group do you belong to?

1. 25-34 years old
2. 35-49 years old
3. 50-64 years old
4. 65 and over
   - - 1. Do you have any other ideas you would like to share about physical activity for people diagnosed with cancer?
